# Enhanced Heterogeneous Fenton Degradation of Organic Dyes by Bimetallic Zirconia-Based Catalysts

**DOI:** 10.3390/molecules29092074

**Published:** 2024-04-30

**Authors:** Eleonora Aneggi, Sajid Hussain, Walter Baratta, Daniele Zuccaccia, Daniele Goi

**Affiliations:** 1 Dipartimento di Scienze AgroAlimentari, Ambientali e Animali, Sezione di Chimica, Università di Udine, e INSTM, 33100 Udine, Italy; walter.baratta@uniud.it (W.B.); daniele.zuccaccia@uniud.it (D.Z.); 2 Dipartimento Politecnico di Ingegneria e Architettura, Università di Udine, e INSTM, 33100 Udine, Italy; sajid.hussain@unipd.it (S.H.); daniele.goi@uniud.it (D.G.); 3 Dipartimento di Ingegneria Industriale, Università di Padova, 35131 Padova, Italy

**Keywords:** heterogeneous Fenton, AOP, dyes, catalyst, transition metals

## Abstract

The qualitative impact of pollutants on water quality is mainly related to their nature and their concentration, but in any case, they determine a strong impact on the involved ecosystems. In particular, refractory organic compounds represent a critical challenge, and several degradation processes have been studied and developed for their removal. Among them, heterogeneous Fenton treatment is a promising technology for wastewater and liquid waste remediation. Here, we have developed mono- and bimetallic formulations based on Co, Cu, Fe, and Mn, which were investigated for the degradation of three model organic dyes (methylene blue, rhodamine B, and malachite green). The treated samples were then analyzed by means of UV-vis spectrophotometry techniques. Bimetallic iron-based materials achieved almost complete degradation of all three model molecules in very short time. The Mn-Fe catalyst resulted in the best formulation with an almost complete degradation of methylene blue and malachite green at pH 5 in 5 min and of rhodamine B at pH 3 in 30 min. The results suggest that these formulations can be applied for the treatment of a broad range of liquid wastes comprising complex and variable organic pollutants. The investigated catalysts are extremely promising when compared to other systems reported in the literature.

## 1. Introduction

The accumulation of non-biodegradable organic compounds in the aquatic system represents a serious threat to water and its biota [1,2,3]. These molecules are products, by-products, or waste from industrial activities. The high stability of these molecules requires targeted actions, aimed at both the mitigation and prevention of the problem, since conventional processes are unable to remove them from polluted matrices [4,5,6,7,8]. Among the most studied persistent organic compounds are dyes, personal care products, and active ingredients of pharmacological origin [9,10,11]. Specifically, dyes are often used as a reference in the development of innovative methods for liquid waste remediation. There is a large variety of advanced oxidation processes (AOPs) for wastewater treatment with proven efficacy [12,13,14,15,16]. Growing research interest has focused on developing alternatives that improve their benefits and reduce their impacts, exploiting the promising potential of these techniques. Each treatment depends on the controlling factors of the reactions, including chemical reagent input, energy, and the overall cost of the process. The choice of the technical specification is therefore based on process optimization and resource management. 

Advanced oxidation processes (AOPs) are a set of chemical treatments designed to remove organic compounds from wastewater and liquid waste. They are based on the in situ production of highly reactive radical species (hydroxyl radicals, •OH); once formed, they non-selectively attack the contaminants according to the diffusion gradient in the solution [17,18,19,20]. AOPs refer mainly to processes that exploit particular reagents to create radicals: ozonation, which uses the ozone molecule (O_3_) [21]; the peroxymonosulfate (PMS) activation with the formation of PMS radicals (SO_5_^•−^) [22,23]; the Fenton and heterogeneous Fenton reactions, which exploit hydrogen peroxide (H_2_O_2_) [24]; and reactions employing physical fields, particularly ultraviolet radiation [25,26,27,28]. Other processes that are not of primary importance for industrial-scale treatment are electrochemical [29], the use of microwaves [30], ultrasound [31], and, finally, hybrid processes [32,33]. Hydroxyl radicals are produced with the help of one or more primary oxidants (such as ozone, hydrogen peroxide, or oxygen) and/or energy sources (such as, for example, ultraviolet light) or catalysts [18]. 

The heterogeneous Fenton process is the preferred alternative among the various AOP techniques, as it allows for the application of advanced oxidation treatments on a large scale in a sustainable way: the use of solid-phase catalysts reduces the problem of the production of large quantities of contaminated sludge, while the careful choice of the catalytic formulation offers the opportunity to operate at less extreme pH values, optimizing the redox cycle [18,34,35,36]. 

**Figure 1:**
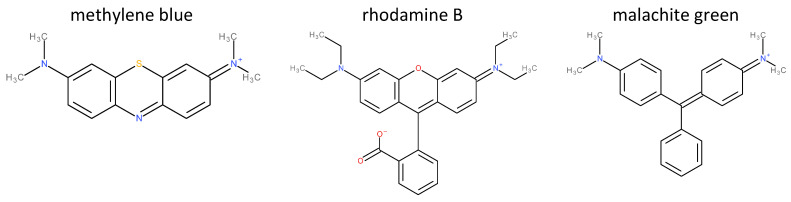
Dyes used in this study.

Previously, we investigated copper-based catalysts, highlighting the promising performance of these formulations [24,37,38,39]. In a recent work [40], we found a synergistic effect of copper and iron in bimetallic catalysts. The developed material not only displays the highest ibuprofen mineralization under optimal conditions but also exploits its activity over a wider pH range (3–5) with extremely low metal leaching. In light of these results, here, we have developed other bimetallic compositions based on Co, Cu, Fe, and Mn to extend the range of effectiveness of the heterogeneous Fenton method in terms of pH and selectivity (tuning catalysts able to degrade a wide range of compounds with high efficiency). Therefore, in this study, two series of catalysts, monometallic and bimetallic, were prepared to investigate their activity and to evaluate the interactions between the combined metals. The experiments were carried out on three different matrices (Figure 1), three dyes widely studied in the literature, due to their high resistance to degradation: methylene blue (MB), rhodamine B (RB), and malachite green (MG) [41,42,43,44,45,46,47]. The three target molecules were chosen due to them being compounds that are very difficult do degrade and need to be treated by advanced oxidation processes to be effectively removed from liquid waste. The different resistance to degradation of the three dyes depends on the intrinsic characteristics of the molecules under consideration and their degree of refractoriness to oxidation in Fenton reactions.

The main purpose of this study is to screen the activity of different catalysts for the degradation of dyes from liquid waste. This is a preliminary study mainly focused on the degradation ability of different catalysts on the three target molecules. The investigation carried out on the model molecules allows for a comparison of the obtained outcomes with the extensive research that already exists for these compounds, and, thanks to the comparison between the measurements carried out and pre-existing knowledge, to draw significant conclusions about the object of this study.

## 2. Results and Discussion

### 2.1. Textural and Structural Characterization

The structural and morphological characterization of the catalysts was carried out by means of surface area measurement (BET) and X-ray powder diffraction (XRD). The nominal composition and BET surface area of the investigated materials are given in Table 1.

**Figure 2:**
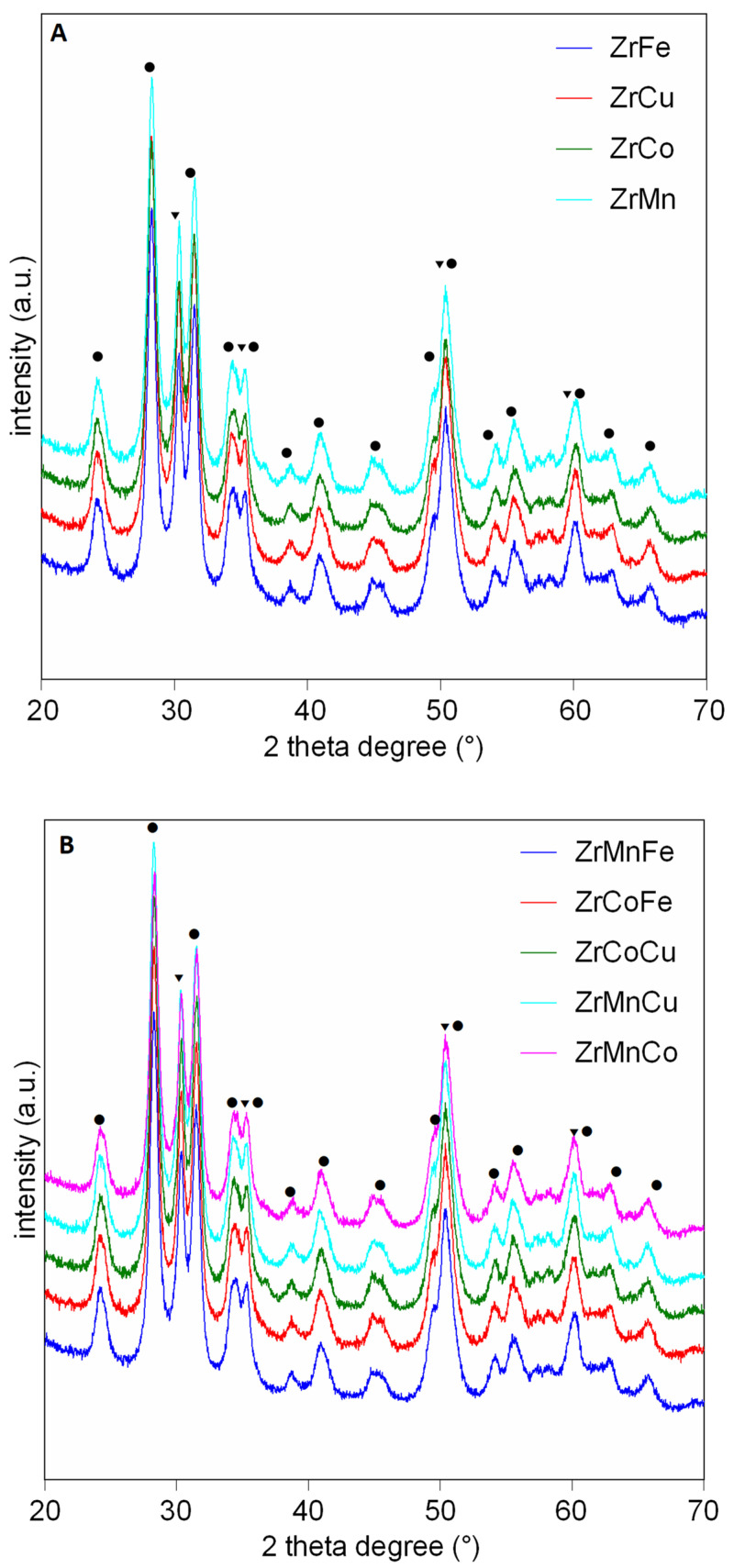
XRD profiles for (**A**) monometallic and (**B**) bimetallic catalysts (●, ZrO_2_ monoclinic; ▼, ZrO_2_ tetragonal).

The surface area of bare ZrO_2_ is 64 m^2^/g. The addition of a single metal does not significantly affect the surface area (53–56 vs. 64 m^2^/g). Even for bimetallic systems, the influence on the surface area is minimal, with a larger decrease found for systems based on Co, i.e., ZrCoCu and ZrCoFe, for which a value of 46 m^2^/g is observed. Figure 2 shows the diffraction profiles of monometallic and bimetallic catalysts supported on zirconia. 

The analysis of the diffraction profiles indicates the presence of two phases of ZrO_2_, one monoclinic and the other tetragonal. No peaks due to the presence of metals supported on zirconia are visible. This may be due, on the one hand, to the low content of metal (2.5%) and, on the other hand, to the probable homogeneous dispersion of the metal on the support. High dispersion is of fundamental importance for the catalytic activity of the materials, since the larger the dispersion, the greater the number of metallic active sites on the surface of the support. A low dispersion should lead to the presence of metal clusters, which would be highlighted by the analysis of the diffraction profiles.

**Figure 3:**
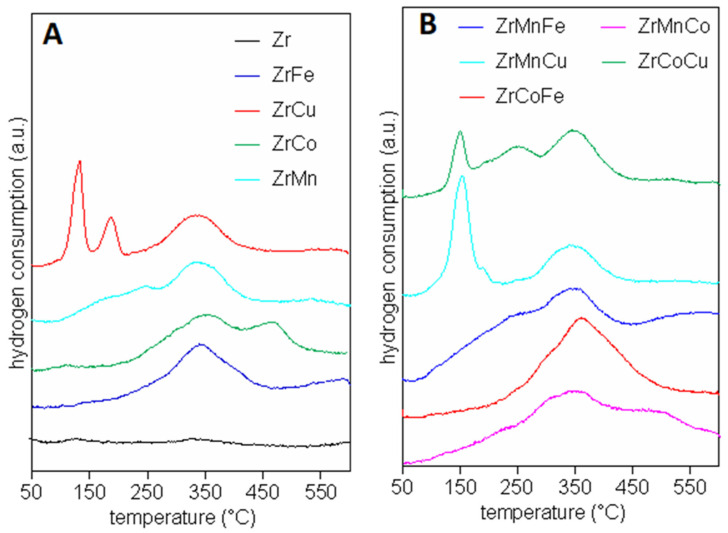
TPR profiles of investigated samples, (**A**) monometallic; (**B**) bimetallic catalysts.

The reducibility of mono- and bimetallic catalysts was also investigated by H_2_ temperature-programmed reduction (Figure 3). Zr support shows a flat signal, a TPR feature of a typical “non reducible” material. 

ZrFe exhibits a very broad peak between 250 and 450 °C, which could be related to free Fe_2_O_3_ on the zirconia surface. It is commonly known that iron oxide undergoes a two-phase reduction of Fe_2_O_3_ to Fe^0^ by passing through Fe_3_O_4_ (magnetite) [48]. ZrCu has three main reduction peaks at 130, 190, and 340 °C due to the reduction of copper species. The three peaks could be related to different types of Cu phase over zirconia. 

The peak at 340 °C can be assigned to the reduction of crystalline CuO. Lower temperature peaks can be attributed to the reduction of highly dispersed Cu^2+^ species, indicating a two-step reduction from Cu^2+^ to Cu^+^ (140 °C) and from Cu^+^ to Cu^0^ (190 °C) [49]. ZrMn exhibits a broad peak centered at 200 °C and another signal at 337 °C. According to the literature, the first reduction step can be associated with the reduction of Mn_2_O_3_ to Mn_3_O_4_, while the second stage can be due to reduction of Mn_3_O_4_ to MnO [50]. Two distinct peaks centered at 350 and 465 °C were detected in the ZrCo profile, corresponding to the reduction of Co_3_O_4_ to CoO and subsequently to Co [51]. The quantitative analysis of the TPR profile (Table 1) indicates a partial reduction of iron species for ZrFe (0.43 mmol H_2_/g_cat_ against 0.67 mmol H_2_/g_cat_ required for the complete reduction of Fe_2_O_3_), while for ZrCo, ZrCu, and ZrMn, the reduction is almost complete (0.43, 0.39 and 0.45 mmol H_2_/g_cat_ required for the complete reduction of Co, Cu, and Mn species, respectively).

The TPR profiles of bimetallic catalysts exhibit peaks due to the to individual metal reduction steps. ZrMnCo shows a broader signal from 150 to 500 °C, including all the peaks evidenced in the monometallic formulations, ZrMn and ZrCo (0.57 mmol/g vs. 087 mmol/g of the theoretical consumption). ZrCoFe displays a larger consumption of hydrogen from 150 to 450 °C, including the peaks due to the reduction of iron and cobalt species. ZrCoFe undergoes a partial reduction (55%) with a consumption of 0.60 mmol H_2_/g_cat_, while the complete reduction required 1.09 mmol/g. Copper-based materials (ZrCoCu and ZrMnCu) both show a signal at 345 °C. In addition, ZrMnCu exhibits a large signal at 155 °C, while ZrCoCu displays a smaller peak at 155 °C and an additional peak at 250 °C. The H_2_ consumption is similar for the two copper-based catalysts, 0.40 for ZrMnCu and 0.46 mmol/g for ZrCoCu, and is lower than the theoretical amount (0.85 and 0.82 mmol/g). ZrMnFe exhibits a very broad peak starting at low temperatures (around 80 °C) up to 450 °C and is the catalyst with larger hydrogen consumption (0.71 mmol/g; 63% reduction to the theoretical amount).

### 2.2. Catalytic Activity

First of all, preliminary tests were carried out to evaluate the effect of the individual reagents on the degradation of organic molecules in solution. Experiments were carried out in the presence of the catalyst alone, without the addition of hydrogen peroxide, with the aim of understanding whether surface adsorption by the catalysts significantly affected the result; then, the experiment was replicated without the addition of catalysts to evaluate if the oxidative capacity of H_2_O_2_ was relevant. In both cases, the results were not significant, with degradation values of less than 10%. Thus, the catalytic activity is due to the combined effect of Fenton-like reagents that include the oxidizing agent (H_2_O_2_) and the catalyst.

#### 2.2.1. Monometallic Catalysts

Monometallic materials were investigated for the degradation of the three dyes at different pH values. Indeed, pH is the most significant parameter affecting the activity of metal catalysts in a Fenton process, since it determines the oxidation states and the processes that can take place. The reaction was studied both at a highly acidic pH (pH 3), for which an excellent response was expected, especially for the iron-containing catalysts, and at a pH more shifted towards neutral values (pH 5). The latter was chosen to investigate the change in catalytic activity at a more sustainable pH in view of a possible large-scale application. The first compound investigated as a target molecule was methylene blue. 

The degradation of the target molecule was investigated by spectrophotometric analysis (Figure S1). The maximum absorbance for methylene blue occurs around 665 nm; the main peak is truncated when the absorbance exceeds the sensitivity of the instrument. Peaks below 350 nm are not due to absorbance phenomena in the visible spectrum but are due to absorptions in the ultraviolet (UV) region. Figure S1A shows a progressive reduction in the main peak due to the Fenton treatment. While ZrMn and ZrCo show only a moderate reduction in the absorbance of the characteristic methylene blue peak, the reduction is significant for ZrCu and is almost complete for ZrFe. In addition, the UV region also displays a significant modification of the peaks for ZrCu and ZrFe samples. The comparison of the spectra at pH 3 (Figure S1A) and pH 5 (Figure S1B) shows a drastic decrease in absorbance and, consequently, a significant increase in the activity for the copper-based catalyst. 

**Figure 4:**
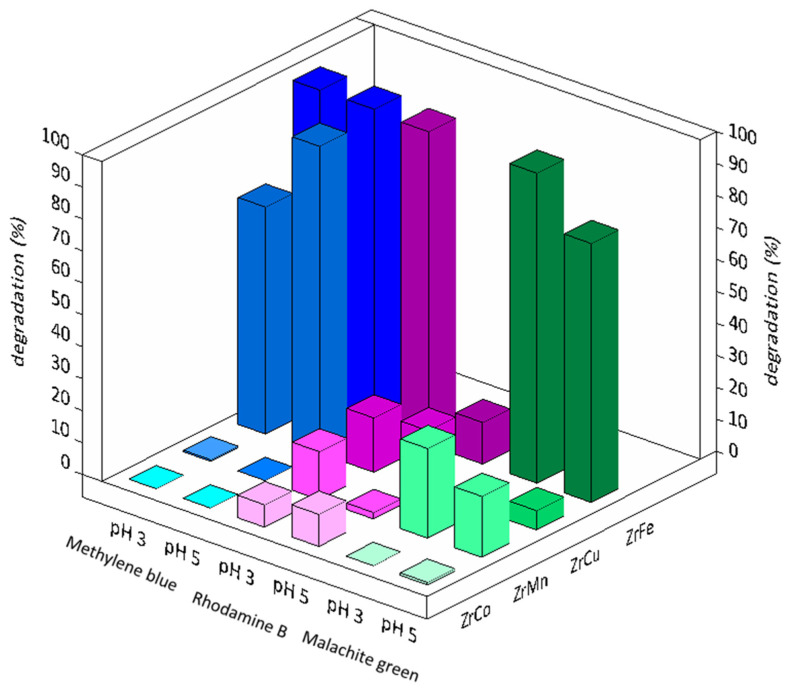
Degradation obtained with Fenton treatment of the three model solutions with monometallic catalysts.

Figure 4 shows the degradation of methylene blue after 30 min of Fenton treatment as obtained by analyzing the absorbance at 665 λ in the UV–visible spectra. ZrFe and ZrCu promoted high degradation, while ZrMn and ZrCo did not degrade the target molecule. In particular, ZrFe achieved almost complete degradation both at pH 3 (99%) and at pH 5 (98%), thus demonstrating that it was not significantly affected by the pH variation. In contrast, copper showed a good response at pH 3 (71%), but achieved a notable increase in activity at pH 5 (96%). This is consistent with the expected higher activity around pH 5–6 for this metal [18,39]. 

The second target molecule investigated was rhodamine B. The maximum absorbance for rhodamine B occurs around 580 nm. The heterogeneous Fenton reaction was tested in a markedly (pH 3) and moderately (pH 5) acidic environment to evaluate the performance of the catalysts both at the ideal pH and at milder and more sustainable conditions with a view to large-scale application. ZrFe is the only catalyst able to efficiently degrade rhodamine B at pH 3 (98%), but the activity is dramatically reduced at pH 5 (12%). The other samples show a low percentage of abatement for both pH conditions, not more than 20%. However, while ZrMn produced better results at pH 3 (15%, versus 3% at pH 5), both ZrCu and ZrCo gave comparable results at different pHs (Figure 3). The copper-based formulation reduced the target molecule by about 18%, while the cobalt-based material reduced it by 8%. The activity of catalysts containing copper and cobalt was slightly affected by pH, while the effect is significant for Mn- and Fe-based materials. Analysis of the UV–visible spectra shows a significant decrease in absorbance only for ZrFe at pH 3 (Figure S1C,D).

The third molecule used in this study was malachite green. The maximum absorbance for malachite green occurs at around 617 nm. The iron-containing catalyst is still the most active, with a degradation percentage of 97% at pH 3. As already occurred in the case of rhodamine B, the activity is strongly influenced by pH and decreases to about 80% when the reaction is carried out at pH 5. The catalysts based on Cu and Co are not active and show a degradation of less than 10%, while ZrMn seems to have moderate activity, 28% at pH 3 and 19% at pH 5. The analysis of the UV–visible spectra exhibits an almost complete reduction in absorbance for ZrFe at both pH levels and a moderate reduction for ZrMn at pH 3 (Figure S1E,F).

ZrFe is the most active catalyst, considering all three dyes. At pH 3, ZrFe provided almost total removal of the molecules from the model solution. At pH 5, it displayed an almost complete degradation for methylene blue and an efficient removal for malachite green (80%) while for rhodamine B, it showed a dramatic collapse in efficiency (10%). In conclusion, the activity of ZrFe on methylene blue was not affected by pH variation; it was moderately affected on malachite green and it showed very high sensitivity on rhodamine B. Methylene blue is the most easily degraded molecule by the investigated catalysts, followed by malachite green and rhodamine B, respectively. Rhodamine B is the most difficult molecule to degrade among the three dyes. The efficiency was generally low with little difference due to pH variation, with the sole exception of ZrFe, which is very active at pH 3. However, as previously reported, ZrFe was very strongly affected by the increase in pH with a significant decrease to around 15%. ZrCu was the only monometallic catalyst that showed a higher degradation at pH 5 compared to the activity at pH 3. This trend is greater for methylene blue compared to the other two dyes. ZrCo was the least active among the monometallic catalysts; it showed no activity with methylene blue and malachite green, and very low degradation (<10%) with rhodamine B. The treatment with ZrMn achieved a very moderate percentage of degradation (20–30%) with the malachite green, while no significant effect was noted with the other two model molecules.

#### 2.2.2. Bimetallic Catalysts

**Figure 5:**
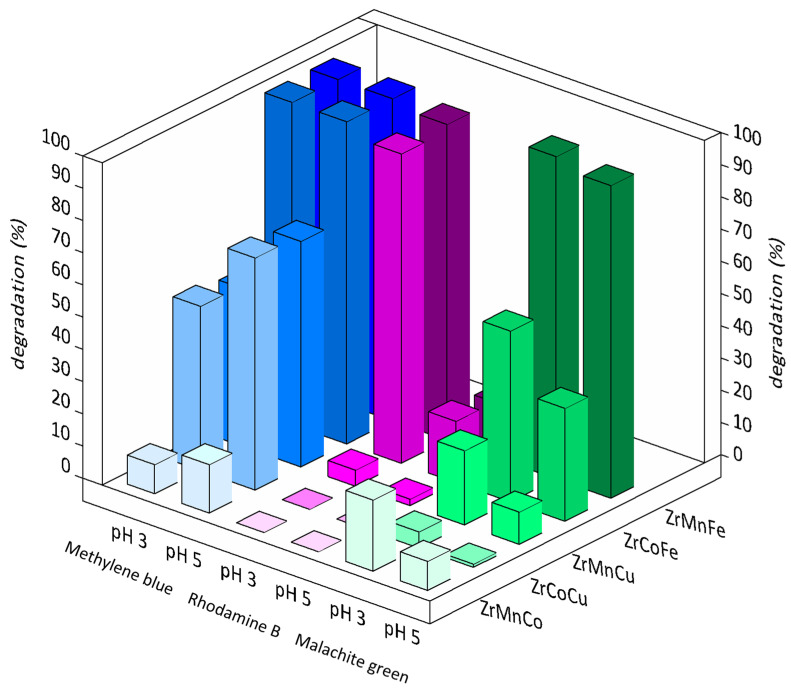
Degradation obtained with Fenton treatment of the three model with bimetallic catalysts.

In order to obtain higher degradation activity, bimetallic catalysts were prepared and tested on the three target molecules at pH 3 and 5 for 30 min. Figure 5 shows the catalytic degradation of the target molecules after catalytic treatment with the investigated formulations (ZrCoCu, ZrCoFe, ZrMnCo, ZrMnCu, ZrMnFe). The degradation efficiency for methylene blue was almost complete for the iron-based catalysts, ZrCoFe and ZrMnFe, at pH 3 and 5. This is consistent with the results obtained with monometallic catalysts, where the catalytic activity of ZrFe was not affected by the pH variation. However, the behavior of the copper-containing bimetallic catalysts is noteworthy: the catalytic activity at pH 3 was high (about 50%), and in agreement with what happened with the monometallic materials, the shift to higher pHs positively influenced the efficiency of the treatment. In particular, ZrCoCu showed a catalytic efficiency of 72% and ZrMnCu of 70%. The combination of manganese and cobalt (ZrMnCo) showed, overall, a low activity, just under 10% degradation at pH 3, with around 15% at pH 5. The analysis of the UV–visible spectra exhibits an almost complete reduction in the absorbance for bimetallic ZrMnFe and ZrCoFe at both pH and a higher reduction at pH 5 for copper-based formulations compared to pH 3 (Figure S2A,B).

For rhodamine B at pH 3, only iron-based materials (ZrCoFe and ZrMnFe) showed very high efficiency, in agreement with the results obtained with the monometallic ZrFe catalyst. All other catalysts did not show sufficient catalytic activity; in fact, ZrMnCu degraded only 6%, while for ZrCoCu and ZrMnCo, the degradation was almost zero. At pH 5, the yield dropped sharply: the degradation efficacies of ZrCoFe and ZrMnFe declined from 100% to 20% (19% for the former, 17% for the latter). In addition, ZrMnCu also decreased from 6% to 2%. ZrCoCu and ZrMnCu were once again confirmed as ineffective. The analysis of the UV–visible spectra exhibits an almost complete reduction in absorbance for the bimetallic ZrMnFe and ZrCoFe only at pH 3 (Figure S2C,D).

For malachite green, the two iron-based catalysts, ZrCoFe and ZrMnFe, achieved the best performance. However, in contrast to what happened with both methylene blue and rhodamine B, the efficiency of ZrCoFe was significantly lower than that of ZrMnFe. Indeed, while ZrMnFe achieves 100% degradation of the target molecule, the efficiency of ZrCoFe stands at around 53%. Paying attention to what happens with copper-containing catalysts, one can appreciate a very different activity between them. ZrCoCu degrades only 5% of the target molecule at pH 3, while ZrMnCu reaches a value almost five times higher (23%). The same performance was recorded for ZrMnCo, which removed 22% of malachite green from the solution. At pH 5, there is only a reduction in efficiency of the performance of each catalyst. At pH 5, ZrMnFe showed a minimal loss of activity (97% of degradation), while a moderate abatement was observed for ZrCoFe (35%). For ZrMnCu and ZrMnCo, it becomes even less than half of what was recorded at a lower pH (10% for ZrMnCu and 9% for ZrMnCo), while the activity of ZrCoCu was negligible, i.e., 1%. The analysis of the UV–visible spectra exhibits an almost complete reduction absorbance for ZrMnFe at both pH, with a moderate reduction for ZrCoFe (Figure S2E,F).

In summary, methylene blue is confirmed as the easiest molecule to degrade: not only did all the catalysts prove to be active but their activity was quite high, with the sole exception of ZrMnCo. As observed for the ZrCu formulation, the degradation of the target molecule is easier at pH 5 compared to pH 3. Rhodamine B, on the other hand, is confirmed as the most difficult compound to degrade. Excellent results are obtained with ZrMnFe and ZrCoFe at pH 3; however, all the other experiments, except for two mediocre results well below the 20% threshold (ZrMnFe and ZrCoFe, at pH 5), show a significant ineffectiveness, regardless of the catalyst used. The catalytic activity on malachite green gave intermediate results between those obtained on the other two model solutions. The trend for monometallic systems is also confirmed: the increase in pH induces a reduction in efficiency.

ZrMnFe is the bimetallic catalyst that achieved the best results; with the sole exception of rhodamine B at pH 5 (20% of degradation), it always reaches an activity higher than 95%. The activity of ZrCoFe on methylene blue and rhodamine B was almost identical to that of ZrMnFe; however, it shows lower activity on malachite green. These materials show very high hydrogen consumption (Table 1) and this feature can be correlated to the higher activity compared to the other formulations. Indeed, the higher reducibility may be related to a more efficient redox cycle, thus enhancing the production of hydroxyl radicals capable of degrading target molecules with high activity. In particular, ZrMnFe achieved better activity for all three model molecules, which may be due to its higher reducibility at low temperatures. Indeed, it shows a broad reduction signal in the range of 50-400 °C. The copper-based catalysts, i.e., ZrMnCu and ZrCoCu, achieved interesting activity only with methylene blue. It is worth noting that the activity increases significantly with increasing pH. This is an important result for the sustainability of the process. ZrMnCo was the least efficient bimetallic catalyst, reaching very low degradation values.

Comparing the results obtained from treatments with monometallic and bimetallic catalysts, iron plays a very important role. Iron was certainly the most active metal; however, the results on malachite green at pH 5 show an interesting synergistic effect with Mn. The combination of iron with cobalt, on the contrary, induces a negative interference for the abatement of malachite green with a decrease in the degradation from 100% to 50% at pH 3 and from 95% to 35% at pH 5. The increase in the efficiency with increasing pH, observed in methylene blue for Cu-based formulations, can be ascribed to the behavior of copper, which is more active at pH 5 (Figure 4). In the case of ZrMnCu and ZrCoCu, when used to degrade methylene blue, the activity was almost exclusively due to copper. Instead, in the experiments on malachite green, the roles were reversed for ZrMnCu: when the contribution of copper disappeared (as observed in Figure 3), ZrMnCu reached approximately 20% degradation thanks to the activity of manganese. However, since the activity of the bimetallic sample is lower compared to bare Mn catalysts on the same solution, it can be concluded that the copper caused a negative interference with the manganese. The activity of ZrCoCu on malachite green was almost zero, since neither of the two metals were proven effective on the target dye. Overall, in metal catalysts supported on ZrO_2_, copper loses activity when coupled with another metal, while iron maintains it, with the sole exception of the treatment of malachite green, for which different behaviors emerge in the presence of manganese (synergistic effect) or cobalt (negative interference), as previously described.

**Figure 6:**
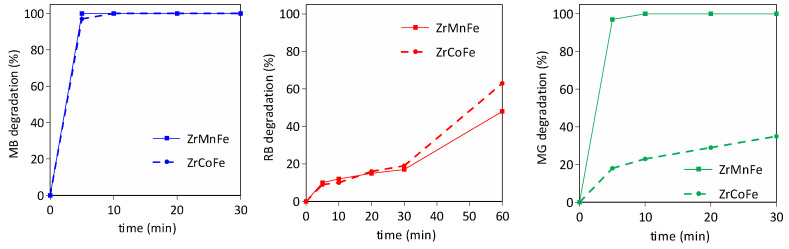
Degradation at different time intervals for the three model solutions (methylene blue (MB), rhodamine B (RB), malachite green (MG)) with ZrMnFe and ZrCoFe at pH 5.

To investigate the activity as a function of time, experiments were carried out with increasing time, from 5 to 30 min. Tests were performed on the two best formulations (ZrCoFe and ZrMnFe) at pH 5 (the most favorable pH from the application perspective). The results are displayed in Figure 6. For methylene blue, a complete degradation was obtained after 5 min; for malachite green, ZrMnFe achieved an almost complete removal, while ZrCoFe attained only 35% abatement. This test highlights that the selected catalysts are significantly active and the degradation process is extremely fast. 

Since rhodamine B was much more difficult to attack and degrade than the other dyes, the reaction was extended to 60 min. At 5 and 10 min, the two catalytic systems showed a very similar degradation of about 10%. Between ten and thirty minutes, the two bimetallic catalysts seemed to reach a plateau situation, where the increase in degradation was almost negligible. In the next half hour, however, they managed to remove up to 63% (ZrMnFe) and 48% (ZrCoFe) of the target molecule from the solution. ZrCoFe and ZrMnFe require longer times to develop their degradation activity in the case of rhodamine B treatment compared to the other dyes.

#### 2.2.3. Comparison with the Literature Results

Methylene blue is a molecule widely used as a target dye in heterogeneous Fenton experiments. Table 2 shows a comparison between the best catalysts obtained in this research and those of the most recent studies. It can be noted that all the catalysts that obtained the highest degradation rates have iron among their components. The bimetallic catalysts developed in this study (ZrMnFe and ZrCoFe) gave remarkable results, since the reaction conditions were advantageous from an application point of view in several key factors. An example is the dose of catalyst used (200 mg/L), which was the lowest with the exception of Fe^0^-Fe_3_O_4_-RGO [52] and CuFe_2_O_4_ [53]. While Fe^0^-Fe_3_O_4_-RGO exhibits high activity, CuFe_2_O_4_ shows the lowest degradation efficiency among the catalysts compared. Since very different concentrations of the target molecule are used in the different studies, it is possible to normalize the data using the ratio between the dose of the catalyst and that of the target molecule. For formulations working in the pH interval of 5–7, this parameter is lower for the catalysts investigated in this study, with the sole exception of Cu-CuFe_2_O_4_/SiO_2_ [54], indicating that a smaller amount of catalyst is needed to degrade the target molecule. Cu-CuFe_2_O_4_/SiO_2_ works with a [cat]/[pollutant] ratio three times lower than our materials and at 25 °C, but required a longer treatment time (120 vs. 5 min) and a higher amount of oxidant (197 vs. 15 mM).

Temperature plays a key role in the activity of homogeneous and heterogeneous Fenton process. Homogeneous systems are generally used at room temperature, but increasing the temperature can improve the oxidation rate and the degree of mineralization by reducing the required amounts of H_2_O_2_ and Fe^2+^. Similarly, heterogeneous catalysts benefit from higher temperatures to achieve higher catalytic activities; indeed, the rate of OH• generation from the oxidant is greatly influenced by the reaction temperature and in turn affects the overall efficacy of the heterogeneous Fenton process. Cu-CuFe_2_O_4_/SiO_2_ [54], which achieved remarkable degradation working at 25 °C at neutral pH and with a low [cat]/[pollutant] ratio, required very high concentration of oxidant compared to our formulation. Cu-CB [48] exhibits 97% degradation at 30 °C but with a higher [cat]/[pollutant] ratio compared to ZrMnFe. FeNi/C-300 [55] and Fe/Acid-MMT [56] work at 45 and 50 °C, respectively, but for longer times and at higher [cat]/[pollutant] ratios compared to our system.

A similar comparison for the treatment of rhodamine B is reported in Table 3. The greater difficulty in degrading this compound is again demonstrated by the reaction conditions reported in the most recent studies: several of the factors controlling the heterogeneous Fenton process are, indeed, more severe compared to what was previously observed in the treatment of methylene blue. The reaction temperatures are higher, on average, as are the doses of catalyst and the doses of H_2_O_2_ used.

The most interesting factor concerns the reaction time: the catalysts analyzed in this study (ZrCoFe and ZrMnFe) were able to almost completely remove the molecule in solution in only 30 min at small doses (200 mg/L) and with low amounts of H_2_O_2_ (15 mmol/L). The most critical parameter was pH, which was the most extreme among those used in recent studies (pH 3 versus 5–7). Some formulations [59,61,62] are able to achieve a higher degradation of rhodamine B at neutral pH but with high catalyst loading (from 250 to 2200 mg/L). As a matter of fact, Fe/Cu-MMT [62] achieves high levels of degradation at 25 °C. With the exception of this formulation, catalysts that perform well at low temperatures require a higher [cat]/[pollutant] ratio and/or more oxidant. Considering the ratio between the concentration of the catalyst and the target molecule, this parameter is largely lower for the catalysts developed in this research, with the only exception of Fe-Cu/γ-Al_2_O_3_ [61], which indicates a lower amount of catalyst necessary to degrade the model pollutant. Fe-Cu/γ-Al_2_O_3_ [61] works with a similar [cat]/[pollutant] ratio, but requires a longer treatment time (210 vs. 30 min) and a higher amount of oxidant (309 vs. 15 mM). It can be concluded that ZrCoFe and ZrMnFe, compared to other formulations in the literature, obtained excellent results in milder conditions in terms of catalyst and oxidant doses and reaction times, but in more extreme pH conditions.

Table 4 shows a comparison between the catalysts used in heterogeneous Fenton reactions on malachite green. The degradation results obtained for this molecule are very high; the result obtained with ZrMnFe (97%) at pH 5 is an excellent outcome. It is the best result obtained without the need to lower the pH to a very acidic condition (pH 3), with the only exception of Cu-NaY [65], which achieved complete degradation at pH 6, but with a high amount of oxidant (175 mmol/L). All the other catalysts were able to remove 100% of the target molecule in solution only at pH 3. Furthermore, ZrMnFe managed to degrade the target molecule in only 5 min, therefore being not only efficient but also very fast.

With the exception of the catalyst composed of zinc, hydroxyhepatite, and magnesium ferrite (Zn/HAP/MgFe_2_O_4_) [66], which degraded 100% of the target molecule in two minutes over a wide range of pH (3–7) at 30 °C, the other systems listed in the table required longer times (30–70 min) to abate malachite green. However, Zn/HAP/MgFe_2_O_4_ works with a very high [cat]/[pollutant] ratio (57) compared to that of ZrMnFe (15). In summary, the good performance obtained for ZrMnFe in this study can be highlighted.

**Table 4:** Comparison of results for malachite green degradation in heterogeneous Fenton reaction.

Catalysts	[cat] (mg/L)	[H_2_O_2_] (mM)	[Pollutant] (mg/L)	[Cat]/[Pollutant]	T (°C)	pH	Time (min)	Degr. (%)	Ref.
ZrMnFe	200	15	13.2	15	70	3	5	100	This work
Cu–NaY	300	175	50	6	60	6	60	100	[65]
Cu-Cu_2_O	300	1	3.65	82	60	3	30	100	[67]
Zn/HAP/MgFe_2_O_4_	571	5	10	57	30	3–7	2	100	[66]
ZrMnFe	200	15	13.2	15	70	5	5	97	This work
Fe_3_O_4_/SiO_2_	15 mg *	50 μL *	25	n.a.	30	6.7	30	96	[68]
Diatomite/MnSiO_3_	400	30	500	0.8	30	n.a.	70	93	[69]

* No information about the volume of solution used is given in the text.

Therefore, it can be concluded that the catalysts developed in this work, particularly ZrMnFe and ZrCoFe, are very promising formulations in heterogeneous Fenton-type treatments since they are able to degrade target molecules with different chemical characteristics with high efficiency. This ability is extremely important, as it indicates that their reaction mechanism is not selective, but enables their use on a wide range of organic pollutants, thus resulting in systems that can be used for liquid waste of different origins. In fact, these systems are very active (degradation greater than 96%) on different target dyes, the oxidation is extremely fast (<30 min), and the conditions are rather mild compared to other systems reported in the literature (especially the dose ratio of catalyst/target molecule and pH). Compared to some promising formulations reported in the literature (Table 2, Table 3 and Table 4) that are active at 25–30 °C, our catalysts work at 70 °C. In general, the higher temperature used in our treatment is balanced by less stringent conditions in terms of the amount of oxidant used, the [cat]/[pollutant] ratio, and the reaction time.

## 3. Materials and Methods

### 3.1. Materials

**Scheme 1:**
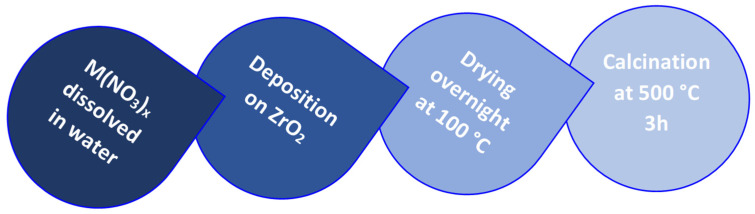
Preparation of monometallic and bimetallic catalysts.

Two series of metal catalysts, monometallic and bimetallic, were prepared on a zirconia (ZrO_2_) support. In the first series, the monometallic catalysts, the zirconia support was modified by the addition of 2.5 wt% of a transition metal (Co, Cu, Fe or Mn), while the bimetallic catalyst series was obtained by impregnation of two metals (Co/Cu, Co/Fe, Co/Mn, Cu/Mn and Fe/Mn) at 2.5 wt% each. The ZrO_2_ support was prepared by calcination of zirconium hydroxide at 500 °C for 3 h (Mel Chemicals, Manchester, UK). The metal precursors (cobalt(II) nitrate hexahydrate Co(NO_3_)_2_•6H_2_O, copper (II) nitrate hemi (pentahydrate) Cu(NO_3_)_2_•2,5H_2_O, iron (III) nitrate nanohydrate Fe(NO_3_)_3_•9H_2_O, manganese (II) nitrate hydrate Mn(NO_3_)_2_•H_2_O; Sigma Aldrich, Saint Louis, MO, USA) were dissolved in a volume of water previously determined by evaluating the wetting point of the zirconia; this aqueous solution was then added to the support for incipient wetness impregnation (IWI). Bimetallic materials were obtained by simultaneous impregnation of two metals. After impregnation, the samples were dried at 100 °C overnight and then calcined in a muffle at 500 °C for 3 h to remove the nitrates, leaving the metal species bonded to the support. The procedure is summarized in Scheme 1.

The surface areas of the samples were measured according to the BET method by nitrogen adsorption at −196 °C, using a Tristar 3000 gas adsorption analyzer (Micromeritics). Structural features of the catalysts were characterized by X-ray diffraction (XRD). A Philips X’Pert diffractometer was used to collect XRD profiles (40 kV and 40 mA using Ni-filtered Cu-Kα radiation) in the range of 20°–80° (step size of 0.02° and a counting time of 20 s per angular abscissa). Phase identification was carried out using the Philips X’Pert HighScore software v. 1.2. Redox behavior was investigated by means of temperature-programmed reduction (TPR) experiments. Samples (40 mg) were pretreated at 500 °C for 1h in air and then heated under a 4.5% H_2_/N_2_ mixture from room temperature to 600 °C in an Autochem II 2920 Instrument (Micrometrics). H_2_ consumption was monitored during the experiments.

### 3.2. Catalytic Activity Measurements

The present study was carried out using three model dyes (Figure 6): methylene blue (Sigma Aldrich, Saint Louis, MO, USA), rhodamine B (Sigma Aldrich, Saint Louis, MO, USA), and malachite green (Sigma Aldrich, Saint Louis, MO, USA). Aqueous solutions were prepared by dissolving the appropriate amount of each dye in ultra-pure water to obtain a concentration of 10 mg/L of organic C.

The reference solution was acidified by progressive addition of H_2_SO_4_ (Sigma Aldrich, Saint Louis, MO, USA), until the desired pH was reached; the catalytic activity was tested at both pH 3 and pH 5.

The dye solution (50 mL) was loaded with the required amount of catalyst (200 mg/L) and heated at 70 °C under reflux and continuous stirring conditions (500 rpm), using an Omni multistage reaction station. Finally, 15 mmol/L of H_2_O_2_ (3% *w*/*w*) was added into the reaction system and the Fenton-like reaction was carried out for 30 min. A hydrogen peroxide solution (3% *w*/*w*) was prepared, starting from 30% *w*/*w* in H_2_O from Sigma Aldrich. At the end of the reaction, samples were centrifuged in an Eppendorf Centrifuge 5804 R (5000 rpm) and filtered through 0.45 µm membrane filters. The oxidation process was optimized by evaluating the effects of pH (3 and 5). The catalytic activity of the investigated systems was determined through spectrophotometric analysis of the UV–visible spectrum (dye degradation). All experiments were conducted in triplicate to verify the reproducibility of our activity measurements, and the resulting errors were within 5% for degradation by means of UV–visible spectroscopy.

## 4. Conclusions

The bimetallic catalysts developed in this study, in particular ZrMnFe and ZrCoFe, show very promising activity for the degradation of dyes by the heterogeneous Fenton process. They remove the target molecules with very high efficiency rates, in reaction conditions that are more advantageous in several aspects than those found in the literature. Specifically, an interesting parameter is the ratio between the dose of the catalyst and the concentration of the pollutant used during the treatment: the value of this ratio was generally always much lower in the experimental tests carried out in this research compared to other studies in the literature. Another very important reaction parameter was the reaction time. This was much lower, especially in the experiments on methylene blue and malachite green, which is of vital importance if a potential large-scale application in treatment plants is evaluated; the low reaction times are therefore a very interesting feature, especially when inserted in the context of a continuous-flow system context, where the time spent in the treatment tanks is relatively short (in the order of tens of minutes). Finally, another aspect of considerable importance concerns the ability of the developed catalysts to degrade different molecules with high activity; these results are interesting for an application of the investigated systems for the abatement of substrates with variable composition, both in terms of the structural complexity of the target organic compounds and in terms of the number of contaminants and their relative concentration. The proposed work is a preliminary investigation of zirconia-based bimetallic formulations for the degradation of organic molecules by a heterogeneous Fenton process. The next step of this work will be the optimization of the proposed catalytic formulations to improve the catalytic performance and their application on more complex matrices, such as model solutions obtained by mixing together more than one dye/organic compound or real liquid wastes.

## Figures and Tables

**Table 1:** Composition and textural characterization of investigated samples.

Name	Nominal Composition	Surface Area (m^2^/g)	H_2_ Consumption (mmol/g)
ZrCo	Co(2.5%)/ZrO_2_	53	0.44
ZrCu	Cu(2.5%)/ZrO_2_	56	0.40
ZrFe	Fe(2.5%)/ZrO_2_	55	0.43
ZrMn	Mn(2.5%)/ZrO_2_	53	0.42
ZrCoCu	Co(2.5%)- Cu(2.5%)/ZrO_2_	46	0.41
ZrCoFe	Co(2.5%)- Fe(2.5%)/ZrO_2_	46	0.60
ZrMnCo	Mn(2.5%)- Co(2.5%)/ZrO_2_	52	0.57
ZrMnCu	Mn(2.5%)- Cu(2.5%)/ZrO_2_	54	0.40
ZrMnFe	Mn(2.5%)- Fe(2.5%)/ZrO_2_	52	0.71

**Table 2:** Comparison of results for methylene blue degradation in heterogeneous Fenton reaction.

Catalysts	[Cat] (mg/L)	[H_2_O_2_] (mM)	[Pollutant] (mg/L)	[Cat]/[Pollutant]	T (°C)	pH	Time (min)	Degr. (%)	Ref.
ZrMnFe	200	15	16.6	12	70	5	5	100	This work
FeNi/C-300	1000	100	30	33	45	7	60	98	[55]
Fe/Acid-MMT	750	0.85	50	15	50	3	30	98	[56]
Fe/Acid-MMT	1000	0.85	50	20	50	6	300	98	[56]
Cu-CuFe_2_O_4_/SiO_2_	200	197	50	4	25	7	120	98	[54]
Fe^0^-Fe_3_O_4_-RGO	100	0.8	50	2	25	3	60	98	[52]
Cu-CB	750	3	20	38	30	6.7	30	97	[57]
ZrCoFe	200	15	16.6	12	70	5	5	97	This work
FeII@MIL-100(Fe)	1000	40	500	2	25	3	300	90	[58]
CuFe_2_O_4_	100	0.5	50	2	25	3	25	74	[53]

**Table 3:** Comparison of results for rhodamine B degradation in heterogeneous Fenton reaction.

Catalysts	[Cat] (mg/L)	[H_2_O_2_] (mM)	[Pollutant] (mg/L)	[Cat]/[Pollutant]	T (°C)	pH	Time (min)	Degr. (%)	Ref.
FeCu@BC_600-2_	200	1	10	20	30	3	60	100	[59]
FeCu@BC_600-2_	250	25	10	25	30	7	360	100	[59]
Cu/SiO_2_	500	29	10	50	60	n.a.	60	100	[60]
Fe-Cu/γ-Al_2_O_3_	2200	309	200	11	70	7	210	100	[61]
ZrMnFe	200	15	14.2	14	70	3	30	98	This work
Fe/Cu-MMT	1500	5	100	15	25	7	90	98	[62]
ZrCoFe	200	15	14	14	70	3	30	96	This work
FeCeO_x_	1500	80	100	15	35	3	150	91	[63]
FeCeO_x_	1500	80	100	15	35	5	150	88	[63]
Mn/Ti-HMS	1000	10	24	42	25	7	100	50	[64]

## Data Availability

Data are contained within the article and Supplementary Materials.

## Supplementary Materials

The following supporting information can be downloaded at https://www.mdpi.com/article/10.3390/molecules29092074/s1: Figure S1: Absorption spectra of model solutions after Fenton treatment with monometallic catalysts. Degradation of methylene blue at (A) pH 3 and (B) pH 5; of rhodamine B at (C) pH 3 and (D) pH 5; and of malachite green at (E) pH 3 and (F) pH 5; Figure S2: Absorption spectra of model solutions after Fenton treatment with bimetallic catalysts. Degradation of methylene blue at (A) pH 3 and (B) pH 5; of rhodamine B at (C) pH 3 and (D) pH 5; and of malachite green at (E) pH 3 and (F) pH 5.
