# Supplementary material for: Enhanced Heterogeneous Fenton Degradation of Organic Dyes by Bimetallic Zirconia-Based Catalysts
Source: Molecules. 2024 Apr 30;29(9):2074. doi: 10.3390/molecules29092074 (PMC11085515; doi:10.3390/molecules29092074)
Supplement: Supplementary file 1 [file molecules-29-02074-s001.zip › molecules-2964264-supplementary.pdf]

# Supplementary Information

# Enhanced heterogeneous Fenton degradation of organic dyes by bimetallic zirconia-based catalysts

**Eleonora Aneggi[1,*], Sajid Hussain[2,3], Walter Baratta[1], Daniele Zuccaccia[1] and Daniele Goi[2]**

[1] Dipartimento di Scienze AgroAlimentari, Ambientali e Animali, Sezione di Chimica, Università di Udine, e INSTM, 33100 Udine, Italy;
[2] Dipartimento Politecnico di Ingegneria e Architettura, Università di Udine, e INSTM, 33100 Udine, Italy;
[3] Dipartimento di Ingegneria Industriale, Università di Padova, 35131 Padova, Italy
* Correspondence: eleonora.aneggi@uniud.it; Tel.: +39-0432-558840

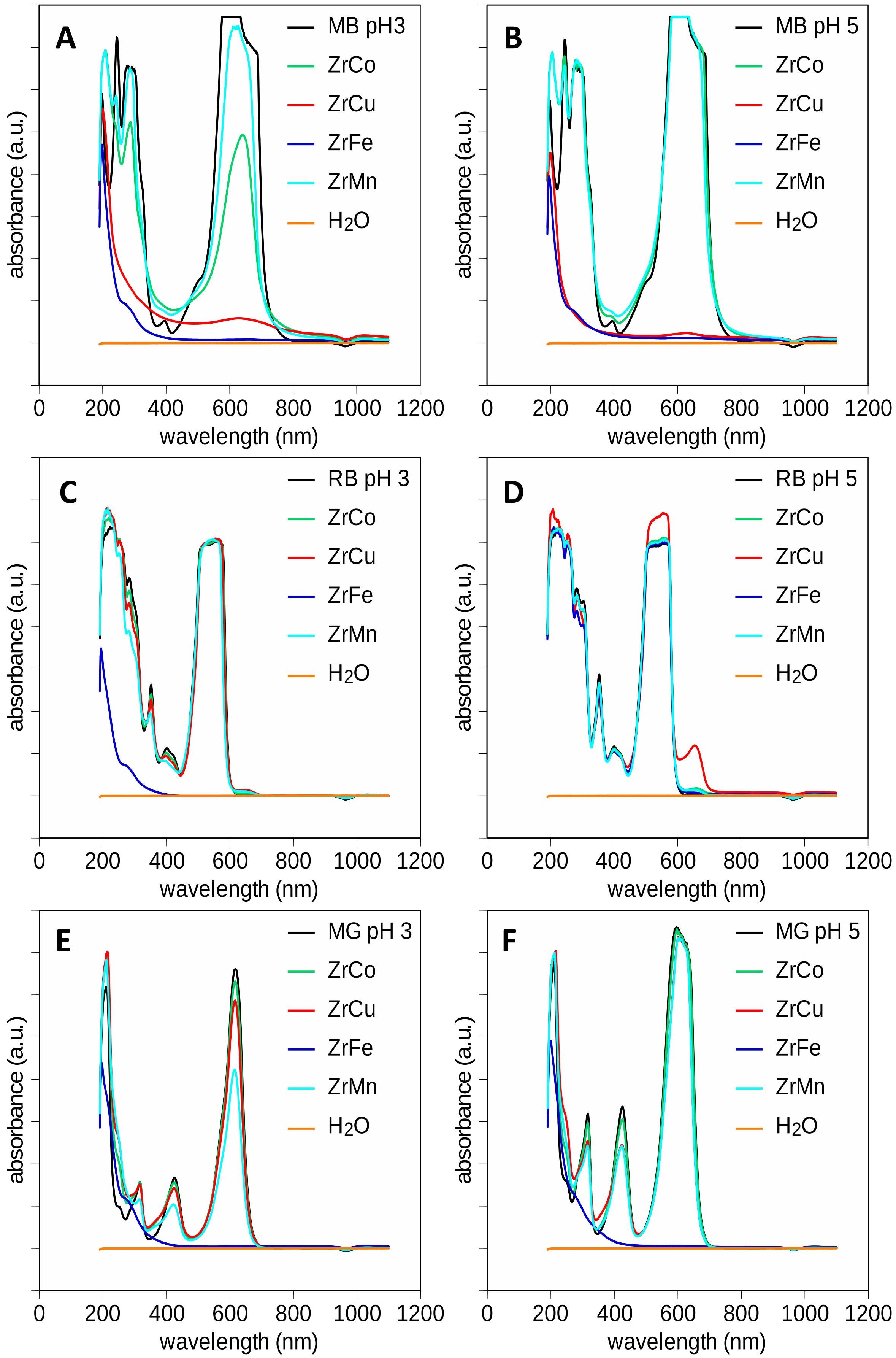


Figure S1. Absorption spectra of model solutions after Fenton treatment with monometallic catalysts. Degradation of methylene blue at A) pH 3 and B) pH 5; of rhodamine B at C) pH 3 and D) pH 5 and of malachite green at E) pH 3 and F) pH 5.

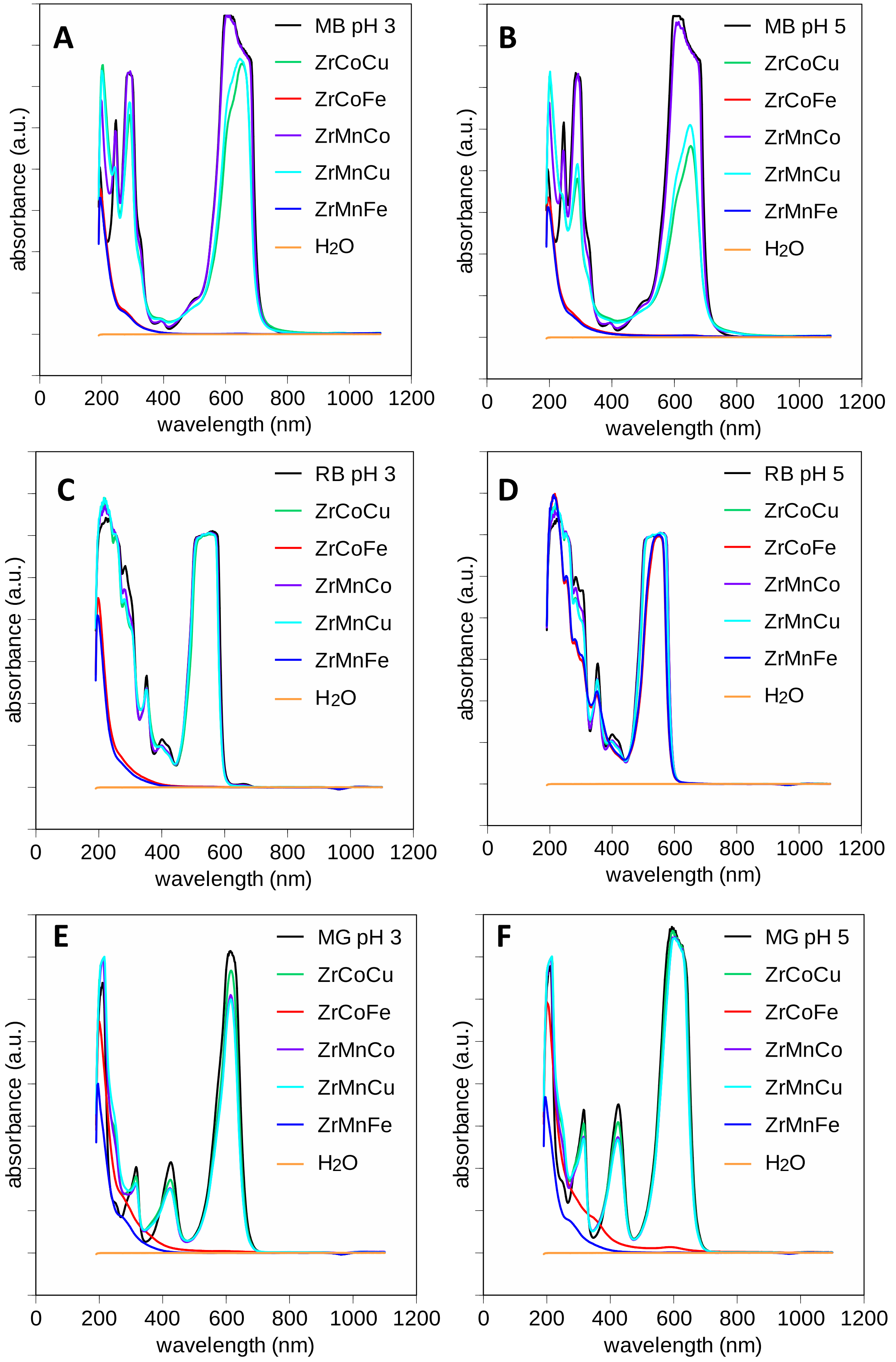


Figure S2. Absorption spectra of model solutions after Fenton treatment with BImetallic catalysts. Degradation of methylene blue at A) pH 3 and B) pH 5; of rhodamine B at C) pH 3 and D) pH 5 and of malachite green at E) pH 3 and F) pH 5
